# Community Assembly of Fungi and Bacteria along Soil-Plant Continuum Differs in a Zoige Wetland

**DOI:** 10.1128/spectrum.02260-22

**Published:** 2022-09-22

**Authors:** Jie Li, Yu-Xuan Liu, Peng-Peng Lü, Yong-Long Wang, Zhong-Feng Li, Yue Zhang, Hui-Yun Gan, Xing-Chun Li, Dipa Mandal, Jing Cai, Zi-Xuan Guo, Hui Yao, Liang-Dong Guo

**Affiliations:** a State Key Laboratory of Mycology, Institute of Microbiology, Chinese Academy of Sciences, Beijing, China; b College of Life Sciences, University of Chinese Academy of Sciences, Beijing, China; c Lushan Botanical Garden, Chinese Academy of Sciences, Jiujiang, China; d Faculty of Biological Science and Technology, Baotou Teacher's College, Baotou, China; e Research Institute of Subtropical Forestry, Chinese Academy of Forestry, Hangzhou, China; Broad Institute

**Keywords:** bacteria, community assembly, fungi, host selection, meta co-occurrence network, source pattern

## Abstract

Distinct plant associated microbiomes live in rhizosphere soil, roots, and leaves. However, the differences in community assembly of fungi and bacteria along soil-plant continuum are less documented in ecosystems. We examined fungal and bacterial communities associated with leaves, roots, and rhizosphere soil of the dominant arbuscular mycorrhizal (AM) plants *Taraxacum mongolicum* and *Elymus nutans* and non-AM plant *Carex enervis* in the Zoige Wetland by using high throughput sequencing techniques. The operational taxonomic unit (OTU) richness of fungi and bacteria was significantly higher in rhizosphere soil than in roots and leaves, and their community compositions were significantly different in the rhizosphere soil, roots, and leaves in each plant species. The co-occurrence network analysis revealed that the sensitive fungal and bacterial OTUs with various taxonomic positions were mainly clustered into different modules according to rhizosphere soil, roots, and leaves in each plant species. Along the soil-plant continuum, the rhizosphere soil pool contributed more source on bacterial than on fungal communities in roots and leaves of the three plant species, and more source on bacterial and fungal communities in leaves of *T. mongolicum* and *E. nutans* compared with *C. enervis*. Furthermore, the root pool contributed more source on bacterial than on fungal communities in leaves of *T. mongolicum* and *E. nutans* but not that of *C. enervis*. This study highlights that the host plant selection intensity is higher in fungal than in bacterial communities in roots and leaves from rhizosphere soil in each plant species, and differs in fungal and bacterial communities along the soil-plant continuum in AM plants *T. mongolicum* and *E. nutans* and non-AM plant *C. enervis* in the Zoige Wetland.

**IMPORTANCE** Elucidating the community microbiome assemblage alone the soil-plant continuum will help to better understand the biodiversity maintenance and ecosystem functioning. Here, we examined the fungal and bacterial communities in rhizosphere soil, roots, and leaves of two dominant AM plants and a non-AM plant in Zoige Wetland. We found that along the soil – plant continuum, host plant selection intensity is higher in fungal than in bacterial communities in roots and leaves from rhizosphere soil in each plant species, and differs in fungal and bacterial communities in the AM- and non-AM plants. This is the first report provides evidence of different assembly patterns of fungal and bacterial communities along the soil-plant continuum in the AM- and non-AM plants in the Zoige Wetland.

## INTRODUCTION

Plant–microbe interactions drive the biodiversity maintenance, community stability, and ecosystem functioning (1, 2). Plant identity and compartment niches affect microbiome communities via host specificity, microenvironment, nutrient supply, and pressure of immune systems (3, 4). In return, microbiomes benefit plant growth and productivity through a variety of mechanisms, including promoting plant nutrient acquisition (5–7) and tolerance to biotic and abiotic stresses, such as pathogens (8, 9) and drought (10, 11). In addition, microbiomes can contribute to carbon and nitrogen cycling in ecosystems through soil organic matter decomposition (7, 12, 13) and atmospheric nitrogen fixation (14, 15). Therefore, elucidating the microbiome assemblage alone the soil-plant continuum will help better understanding the biodiversity maintenance and ecosystem functioning.

Disentangling the interactions among co-occurring organisms using ecological network analysis could provide new insights into the mechanisms underlying species coexistence, community assembly, and stability (16, 17). Topological properties such as modularity and average degree can present the complexity and connectedness among members in co-occurrence networks (18). Because fungi and bacteria have potential interactions with each other, using cross-kingdom co-occurrence network analysis can visualize the co-occurrence patterns and reveal potential connections of fungi and bacteria as an entirety (19). Furthermore, from the perspective of comparative microbiome, microbial groups are sensitive to specific factors may possess specific network properties in co-occurrence network, and those sensitive groups could significantly influence the microbial community composition (16, 20). For example, the sensitive microorganisms mapped into the cross-kingdom co-occurrence network are agglomerated according to management type and/or tillage intensity, and their distribution in the network partially reflected the drivers of community dissimilarity. Specifically, soil bacteria were generally driven by differences in tillage regimes, whereas both management type and tillage were influential for the soil fungi (19). Therefore, the specific fungal and bacterial groups which are sensitive to host plants and/or inhabited compartment niches may possess specific properties in the cross-kingdom co-occurrence network, and the occurrence patterns of which could explain their difference on microbial community, consequently, have ecological importance in community assembly (19, 20).

Previous studies have shown that microbiome communities in compartment niches (e.g., rhizosphere soil, root, and leaf) are different and affected by abiotic factors, such as soil, climate, and spatial distance (21–27). In addition, the assembly of microbiome communities in different compartments could be a consequence of host selection, as the microorganisms require the capability to overcome the host immune system and adapt to the microhabitats shaped by plant skeleton and metabolism in a specific niche (28–31). Soil is assumed to be the largest habitat for diverse microorganisms in terrestrial ecosystems and serves as a rich microbial reservoir for host selection (32, 33). Microbiomes in bulk soil firstly transfer to rhizosphere soil, and then to plant aerial tissues via roots mediated by host selection (26, 27, 34–38). Furthermore, the host selection intensity may be higher on fungi than on bacteria along the soil-plant continuum in ecosystems, as fungi have stronger host preference and linked more tightly to plants than bacteria (37, 39–43). For example, source tracking analysis showed that higher proportion of endophytic bacterial than fungal communities in roots was derived from rhizosphere soil in *Cannabis sativa* (27) and in Zea mays, Triticum aestivum, and *Hordeum vulgare* (26, 38), although higher proportion of endophytic fungal than bacterial communities in leaves was derived from roots in *C. sativa* (27), and higher proportion of fungal than bacterial communities in rhizoplane was derived from rhizosphere soil in *Z. mays*, *T. aestivum*, and *H. vulgare* (26, 38). Therefore, the differences in community assembly of bacteria and fungi along soil-plant continuum need to be investigated in ecosystems.

In ecosystems, soil fungi form mycorrhizas with most terrestrial plant species (44), which can influence the other fungal and bacterial communities in rhizosphere soil, roots, and leaves (45–50), as mycorrhizal fungi can interact with other fungi and bacteria via nutrient redistribution and space competition (51–53). For example, previous studies showed that arbuscular mycorrhizal (AM) fungi affected the bacterial community composition in rhizosphere soil of *Allium porrum*, *Cucumis sativus*, *Trifolium subterraneum* and *Z. mays* (45, 50), leaves of *Deschampsia flexuosa* (47)), and roots of *Allium fistulosum* (49), as well as fungal community composition in rhizosphere soil of *Z. mays* (50). Another study found that AM fungi acted as a major factor in determining the bacterial community composition in plant roots in an upland grazed grassland at Fasset Hill (46). However, the differences in community assembly of fungi and bacteria alone the soil-plant continuum in AM and non-AM plants in ecosystems are less documented.

Zoige wetland is a representative alpine wetland ecosystem on the Qinghai-Tibet plateau in China, with high plant species diversity and important carbon sink function (54). Previous studies have mainly focused on plant diversity and productivity (55, 56), carbon and nitrogen cycling (57, 58), ecosystem conservation (59–61), methane oxidation and flux mediated by functional microbes such as methanotrophs (52, 62, 63), and soil microbial diversity and community composition (64, 65) in the Zoige Wetland. However, how host plant shapes the assembly of microbiome community along the soil–plant continuum in wetlands remains largely unknown. Therefore, we examined the fungal and bacterial communities in rhizosphere soil, roots, and leaves of the dominant AM plants *Taraxacum mongolicum* and *Elymus nutans* and non-AM plant *Carex enervis* in Zoige Wetland using Illumina MiSeq sequencing techniques. We hypothesize that (i) compartment niche affect the community assembly of fungi and bacteria, (ii) plant has higher selection intensity on fungal than on bacterial communities along the soil–plant continuum, and (iii) AM plant has higher selection intensity on fungal and bacterial communities compared with non-AM plant in the Zoige Wetland.

## RESULTS

### Characterization of Illumina sequencing data.

After controlling for sequence quality, 4,194,304 ITS2 sequences were obtained from 5,525,952 raw sequences and clustered into 4,919 non-singleton OTUs at a 97% similarity level. Of these OTUs, 3,308 were identified as fungal. As the fungal sequence numbers ranged from 9,272 to 811,367 among 162 samples, the sequence number was normalized to 9272, resulting in the normalized data set comprised 3,308 fungal OTUs (Additional file, Table S1). The fungal community was dominated by Dothideomycetes (29% of the total fungal sequences), Sordariomycetes (28%), and Leotiomycetes (15%) (Fig. S1a).

A total of 16,239,262 16S sequences were obtained from 19,819,566 raw sequences and clustered into 15,724 non-singleton OTUs at a 97% sequence similarity level. Of these OTUs, 9,272 were identified as bacterial. As the bacterial sequence numbers ranged from 1,943 to 90,681 among 162 samples, the sequence number was normalized to 1,943, resulting in the normalized data set comprised 9,272 bacterial OTUs (Table S2). The bacterial community was dominated by phyla Proteobacteria (52% of the total bacterial sequences), Actinobacteria (24%), and Acidobacteria (9%) (Fig. S1b). For both fungi and bacteria, rarefaction curves for the observed OTUs in the 3 plant species showed no signs of reaching asymptotes, suggesting that further sampling would recover more OTUs (Fig. S2).

### Richness of fungi and bacteria.

**FIG 1:**
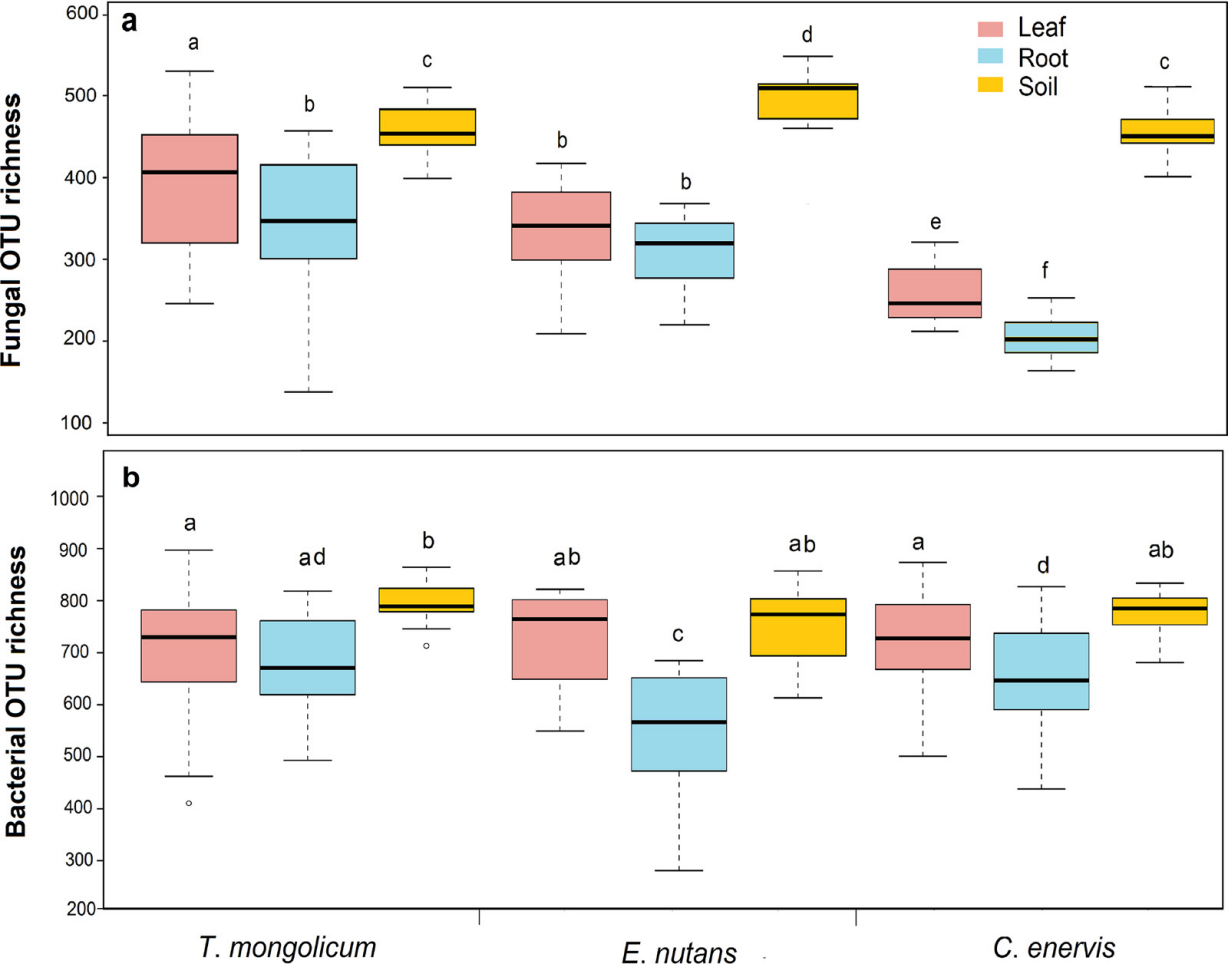
Operational taxonomic unit (OTU) richness of fungi and bacteria associated with 3 plant species. The black line inside each box represents the median value. Bars with different letters indicate significant difference of fungal and bacterial OTU richness respectively, determined by Tukey’s HSD test at *P < *0.05. *T*. *mongolicum*: *Taraxacum mongolicum*; *E*. *nutans*: *Elymus nutans*; *C. enervis*: *Carex enervis*.

One-way ANOVA revealed that compartment niche had significant effect on the OTU richness of bacteria and fungi in *T. mongolicum* (*F *= 8.816, *P < *0.001; *F *= 6.954, *P = *0.002), *E. nutans* (*F *= 73.668, *P < *0.001; *F *= 14.372, *P < *0.001) and *C. enervis* (*F *= 257.653, *P < *0.001; *F *= 7.951, *P = *0.001) (Fig. 1a and b). For example, the fungal OTU richness was significantly higher in rhizosphere soil than in leaves and roots in the 3 plant species (Fig. 1a). Furthermore, the bacterial OTU richness was significantly higher in soil than in leaves and roots in *T. mongolicum*, and higher in rhizosphere soil and leaves than in roots in *E. nutans* and *C. enervis* (Fig. 1b).

### Community composition of fungi and bacteria.

The heatmap revealed that the abundant fungal and bacterial OTUs (top 50) in relative abundance bias occurred in the leaves, roots, and rhizosphere soil of each plant species (Fig. S3a and b). We also found that a small proportion of the total number of fungal and bacterial OTUs was unique in leaves (fungal ≤ 13%, bacterial ≤ 11.8%), roots (fungal ≤ 8.4%, bacterial ≤ 11.4%), and in soil (fungal ≤ 17.9%, bacterial ≤ 10.2%) in 3 species (Fig. S4a and b).

Dothideomycetes were abundant in leaves of *T. mongolicum* and *E. nutans*, and in soils of *E. nutans* and *C. enervis*. Sordariomycetes were abundant in soil of *T. mongolicum* and in leaves of *E. nutans* and *C. enervis* (Fig. S5a). Leotiomycetes were abundant in roots of the three plant species (Fig. S5a). The bacterial phyla Proteobacteria, Actinobacteria, and Acidobacteria were dominated in rhizosphere soil, roots, and leaves of each plant species (Fig. S5a).

**FIG 2:**
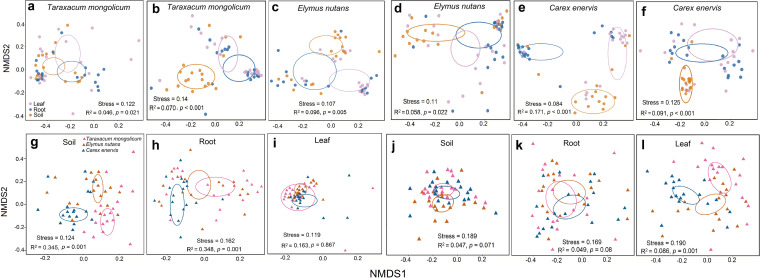
Non-metric multidimensional scaling (NMDS) ordination of the community composition of fungi and bacteria associated with three plant species. The permutational multivariate analysis of variance (PerMANOVA) showed the effect of compartment niche (soil, root, and leaf) and plant identity on the community composition of fungi and bacteria. (a) to (f) Fungi and bacteria in compartment niches in *Taraxacum mongolicum* (a) and (b), *Elymus nutans* (c) and (d) and *Carex enervis* (e) and (f), respectively. (g) – l Fungi and bacteria in soil (g) and (j), root (h) and (k) and leaf (i) and (l) of 3 plant species, respectively.

The PerMANOVA and NMDS ordination indicated that compartment niche significantly affected the community composition of both fungi and bacteria in *T. mongolicum* (*R^2^* = 0.046, *P = *0.021; *R^2^* = 0.070, *P < *0.001) (Fig. 2a and b), *E. nutans* (*R^2^* = 0.096, *P = *0.005; *R^2^* = 0.058, *P < *0.022) (Fig. 2c and d) and *C. enervis* (*R^2^* = 0.171, *P < *0.001; *R^2^* = 0.070, *P < *0.001) (Fig. 2e and f). Furthermore, the fungal community was significantly different in rhizosphere soil (*R^2^* = 0.345, *P = *0.001) (Fig. 2g) and roots (*R^2^* = 0.348, *P = *0.001) (Fig. 2h), but not in leaves among *T. mongolicum*, *E. nutans* and *C. enervis* (*R^2^* = 0.163, *P = *0.867) (Fig. 2i). By contrast, the bacterial community was significantly different in leaves (*R^2^* = 0.086, *P = *0.001) (Fig. 2j), but not in rhizosphere soil (*R^2^* = 0.047, *P = *0.071) (Fig. 2k) and roots (*R^2^* = 0.049, *P = *0.080) (Fig. 2l) in the 3 plant species.

### Co-occurrence network of fungi and bacteria.

**FIG 3:**
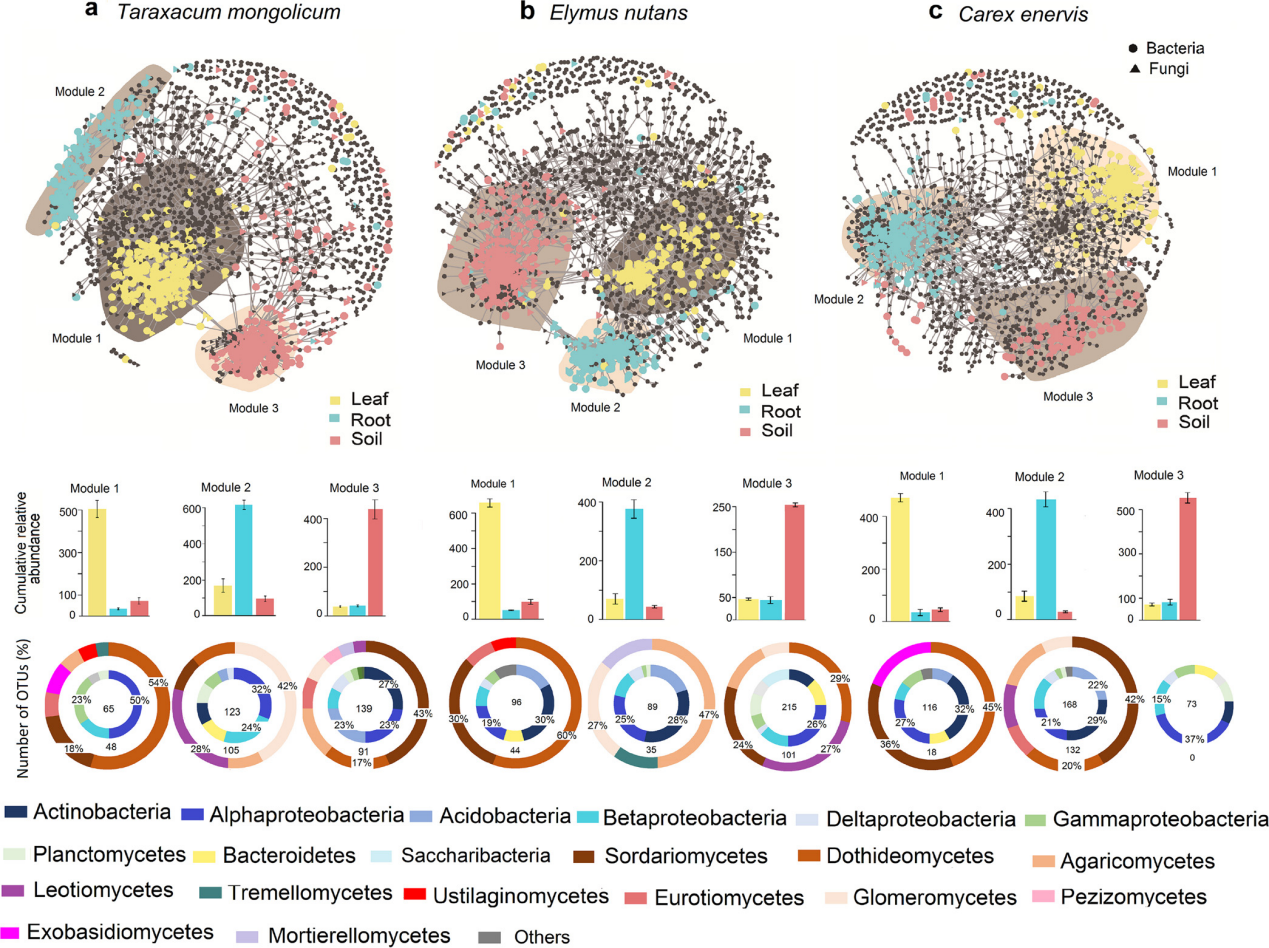
Co-occurrence network structure of fungal and bacterial operational taxonomic units (OTUs) in the leaf, root and rhizosphere soil of *Taraxacum mongolicum* (a), *Elymus nutans* (b) and *Carex enervis* (c). In the network, fungal and bacterial OTUs marked by circle and triangle, respectively. Sensitive fungal and bacterial OTUs to different compartment niches are magnified and colored by yellow, blue, and red, and insensitive bacterial and fungal OTUs are colored by black. Shaded areas in the networks represent the main modules containing majority of sensitive OTUs. In bar graphs, the cumulative abundance of the sensitive OTUs to specific compartment niches marked by different colors in each module. In annulus figures, the percentage of the number of sensitive fungal OTUs in a class to the total number of fungal OTUs in the module (outer annulus) and the number of the sensitive bacterial OTUs in a phylum to the total number of bacterial OTUs in the module (inner annulus) marked by different colors in each module. The total number of sensitive OTUs is showed in the center of annulus.

The cross-kingdom microbial co-occurrence network showed that the sensitive fungal and bacterial OTUs were mainly clustered in three major modules according to rhizosphere soil, root, and leaf of each plant species (Fig. 3a to c). For example, most sensitive fungal and bacterial OTUs specific to rhizosphere soil were mainly distributed in module 1, which mainly comprised members of Actinobacteria and Sordariomycetes in *T. mongolicum*, Dothideomycetes, Leotiomycetes, and Alphaproteobacteria in *E. nutans*, and Alphaproteobacteria in *C. enervis* (Fig. 3a, b, and c, and Table S3). Most sensitive fungal and bacterial OTUs specific to roots mainly distributed in module 2, which mainly comprised members of Glomeromycetes and Alphaproteobacteria in *T. mongolicum*, Agaricomycetes, Actinobacteria, and Alphaproteobacteria in *E. nutans*, and Sordariomycetes and Actinobacteria in *C. enervis* (Fig. 3a, b, and c, and Table S3). Most sensitive fungal and bacterial OTUs specific to leaves mainly distributed in module 3, which mainly comprised members of Dothideomycetes and Alphaproteobacteria in *T. mongolicum*, Dothideomycetes and Actinobacteria in *E. nutans*, and Actinobacteria in *C. enervis* (Fig. 3a, b, and c, and Table S3). Furthermore, among the network parameters, more nodes and edges, higher average degree, higher degree of centralization and connectance, and lower modularity were observed in *C. enervis* compared to *T. mongolicum* and *E. nutans* (Fig. 3a, b, and c, and Table S4).

### The host selection processes and potential sources of fungi and bacteria.

**FIG 4:**
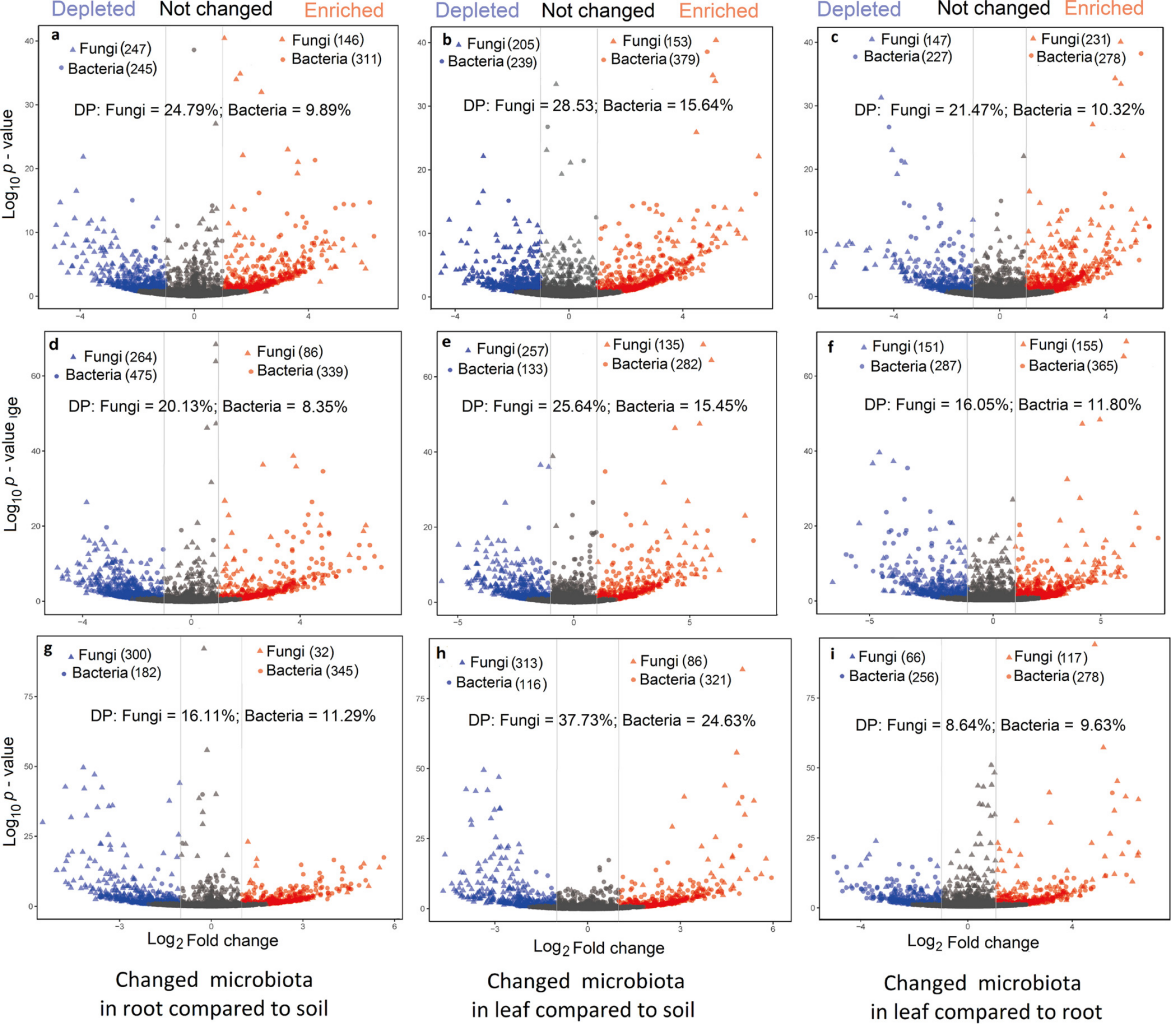
The enriched and depleted operational taxonomic units (OTUs) of fungi and bacteria along soil-plant continuum in the 3 species. (a), (b), and (c) Enriched and depleted OTUs in *Taraxacum mongolicum*. (d), (e), and (f) Enriched and depleted OTUs in *Elymus nutans.* (g), (h), and (i) Enriched and depleted OTUs in *Carex enervis*. Triangles and circles represent fungal and bacterial OTUs, respectively. Enriched OTUs marked by blue, depleted OTUs by red, and no changed OTUs by gray. DP: Proportion of depleted OTUs.

Indicator species analysis and likelihood ratio test showed that certain bacterial and fungal OTUs with various taxonomic positions were enriched or depleted in leaves and roots from rhizosphere soil in the 3 plant species (Fig. 4a to i, and Table S5). Furthermore, compared to rhizosphere soil, the DP values of bacterial and fungal OTUs were lower in roots than in leaves (DP_bacteria_ = 8.35 – 11.29% versus 15.45 – 24.63%; DP_fungi_ = 16.11 – 21.47% versus 25.64 – 37.73%) in the 3 plant species (Fig. 4a, b, d, e, g, and h). Compared to roots, the DP values of bacterial and fungal OTUs were higher in *T. mongolicum* (10.32% and 21.47%; Fig. 4c) and *E. nutans* (11.80% and 16.05%) (Fig. 4f) than *C. enervis* (9.63% and 8.64%) (Fig. 4i).

**FIG 5:**
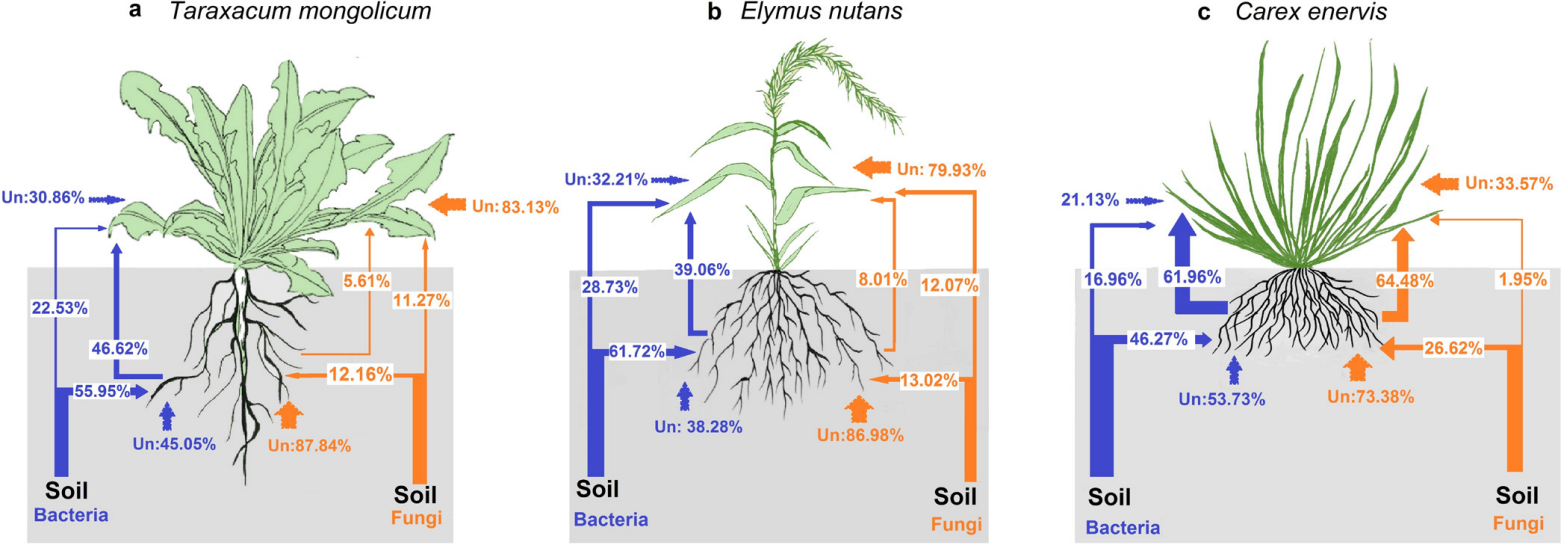
Sources of fungal and bacterial communities along the soil – plant continuum in *Taraxacum mongolicum* (a), *Elymus nutans* (b) and *Carex enervis* (c). Fungi marked by orange lines. Bacteria marked by blue lines. Line thickness is equivalent to the proportion of source contribution. Un: the unknown source.

The source tracker analysis showed that the proportion of bacterial and fungal OTUs was 55.95% and 12.16% in roots derived from rhizosphere soil, 46.62% and 5.61% in leaves from roots, and 22.53% and 11.27% in leaves from rhizosphere soil in *T. mongolicum*, respectively (Fig. 5a). In *E. nutans*, the proportion of bacterial and fungal OTUs was 61.72% and 13.02% in roots derived from rhizosphere soil, 39.06% and 8.01% in leaves from roots, and 28.37% and 12.07% in leaves from rhizosphere soil (Fig. 5b). In *C. enervis*, the proportion of bacterial and fungal OTUs was 46.27% and 26.62% in roots derived from rhizosphere soil, 61.96% and 64.48% in leaves from roots, and 16.96% and 1.95% in leaves from rhizosphere soil (Fig. 5c). Overall, these results suggest that plant has stronger selection intensity on fungal than on bacterial communities in leaves from roots and rhizosphere soil and in roots from rhizosphere soil in the 3 plant species, except for in leaves from roots in *C. enervis*. Furthermore, compared with the non-AM plant *C. enervis*, the AM plants *T. mongolicum* and *E. nutans* had higher host selection intensity on fungal and bacterial communities in leaves derived from roots and on fungal community in roots derived from rhizosphere soil, but lower host selection intensity on fungal and bacterial communities in leaves and bacterial community in roots derived from rhizosphere soil.

## DISCUSSION

The first hypothesis was supported by our findings that the community composition of bacteria and fungi was significantly different in rhizosphere soil, roots, and leaves in each plant species. This may be ascribed to specific microenvironment in different compartment niches (66, 67). For example, the microorganisms in rhizosphere soil are mainly affected by soil pH, mineral elements, organic matter, and root exudates of plants (68), while that in roots and leaves are mainly affected by mechanical characteristics and nutrients supply by host plant (15, 69). Consistently, we identified sets of fungal and bacterial OTUs are specifically sensitive to soil, roots and leaves, which were mainly distributed according to the compartment niches in the cross-kingdom co-occurrence networks, suggesting compartment niche as an important driver of the richness and community composition dissimilarity of fungi and bacteria (18, 19, 26, 38). Furthermore, we found that the community composition of fungi was significantly different in rhizosphere soil and roots, while that of bacteria was different in leaves between AM plants *T. mongolicum* and *E. nutans* and the non-AM plant *C. enervis*, suggesting that the microbial community composition in different compartment niches was affected by AM fungi. This was consistent with previous studies that the AM fungi affected the fungal community composition in rhizosphere soil of *Z. mays* (50) and bacterial community composition in leaves of *Deschampsia flexuosa* (47). Moreover, the mutual preference of AM fungi and their hosts (36, 37, 40) may simplify the complexity of the co-occurrence network of plant associated fungi and bacteria, because the co-occurrence network was more complicate, represented by more nodes and edges, higher average degree and connectance but lower modularity (18, 57, 70, 71) in the AM plants *T. mongolicum* and *E. nutans* than the non- AM plant *C. enervis*. Nevertheless, it needs further validation with more plant species.

The second hypothesis was partially supported by our findings that plant had stronger selection intensity on fungal than on bacterial communities along the soil-plant continuum in the 3 plant species, except for a similar selection intensity on fungal and bacterial communities in leaves from roots in *C. enervis*. Similarly, a previous study showed that lower host selection intensity resulted for higher proportion of endophytic bacterial than fungal communities in roots was derived from rhizosphere soil in *C. sativa*, *Z. mays*, *T. aestivum*, and *H. vulgare* (26, 38). The higher host selection intensity on fungal than on bacterial communities along the soil-plant continuum was presented as lower source contribution (1.95% – 26.62% versus 16.96% – 61.72%) but higher proportion of depleted OTUs (16.11% – 37.73% versus 8.35% – 24.63%) of fungi compared to bacteria. This may be because fungi have stronger host preference than bacteria (35–37, 39, 40). In addition, fungi are linked more tightly to plants than are bacteria, as some fungi can form biotrophic interactions with plants (41, 43), while bacteria tend to have less direct connection to plants (42). Besides, the distinct host selection and source contribution on plant associated fungi and bacteria may depend on the different mechanisms of their community assembly, in that bacteria are strongly structured by stochastic process (72, 73), while fungi are mainly shaped by deterministic process (74, 75). However, the similar host selection intensity on fungal and bacterial communities in leaves from roots in *C. enervis* was presented as almost similar source contribution (64.48% versus 61.96%) and proportion of depleted OTUs (9.63%% versus 8.35%) of fungi compared to bacteria. This may be because there are no AM fungi in roots of *C. enervis*, the host selection on fungal and bacterial communities in leaves from roots may be mainly affected by host selection from functional traits such as leaf area and nutrients, leaf skeleton, metabolic, and immune systems (28, 29, 76, 77), which gave similar host pressure on community assemblies of fungi and bacteria (15, 31, 78).

The third hypothesis was also partially supported by our findings that the AM plants *T. mongolicum* and *E. nutans* had higher selection intensity on fungal and bacterial communities in leaves derived from roots and on fungal community in roots from rhizosphere soil, but lower selection intensity on fungal and bacterial communities in leaves derived from rhizosphere soil and on bacterial community in roots from rhizosphere soil compared with the non-AM plant *C. enervis*. The higher host selection intensity of AM plants on fungal and bacterial communities in leaves derived from roots may be because most of symbiotic AM fungi living in roots do not likely to colonize in leaves, leaving only a few fungi arrived to leaves via roots, and less fungi also reduced the transmission of bacteria to leaves as fungal hyphae acting as habitat and transport vectors for bacterial dispersal and access to substrates (79, 80) compared to the non-AM plant. By contrast, the lower selection intensity of *T. mongolicum* and *E. nutans* on fungal and bacterial communities in leaves derived from rhizosphere soil may be because the different functional characteristics of leaves (15, 31, 78), such as larger leaf area but less hardness allows more microbiomes to occupy and colonize compared to *C. enervis*. In addition, the higher host selection intensity of AM plants on fungal community in roots from rhizosphere soil may be because of restrict screening on AM fungal groups to form mycorrhizal symbiont with roots but not in non-AM plant (36, 37, 40). By contrast, the lower host selection intensity of AM plants *T. mongolicum* and *E. nutans* on bacterial communities in roots from rhizosphere soil may be resulted from more habitat and nutrients provided in hyphosphere for bacteria by the AM fungi (81–84) compared with the non-AM plant *C. enervis*.

### Conclusions.

This study demonstrated that compartment niche plays significant roles in assembly of fungal and bacterial communities. Along the soil-plant continuum, the plant had stronger selection intensity on fungal than on bacterial communities, except in leaves from roots in *C. enervis.* Furthermore, the AM plants *T. mongolicum,* and *E. nutans* had higher selection intensity on community assembly of fungi and bacteria in leaves from roots, and on fungal community in roots from rhizosphere soil compared with the non-AM plant *C. enervis*. On the contrary, the non-AM plant *C. enervis* had stronger selection intensity on fungal and bacterial communities in leaves and roots from rhizosphere soil compared with the AM plants *T. mongolicum* and *E. nutans*. These findings provided evidence of different assembly patterns of plant associated fungi and bacteria along the soil-plant continuum in wetland ecosystem.

## MATERIALS AND METHODS

### Study site and sampling.

The study was carried out in the Zoige national nature reserve on the Qinghai-Tibet plateau (33°25'−34° 80'N, 102° 29'−102°59'E, 3,365 m above sea level). The site has a plateau cold temperate humid monsoon climate, with a mean annual temperature of 1.1°C and a mean annual precipitation of 660 mm (55). The site begins to freeze in late September and is completely thawed in mid-May (55). Plant species in this site are dominated by *T. mongolicum*, *E. nutans*, *C. enervis*, and *Blysmus sinocompressus*, followed by *B. sinocompressus*, *Potentilla anserine*, and *Leontopodium wilsonii* (55). The soil pH was 7.838, the total carbon, total nitrogen, and total phosphorus were 111.9, 8.629 and 1.036, respectively (64).

In June 2019, we randomly selected 18 individuals (replicates) of each of the dominant species *T. mongolicum*, *E. nutans*, and *C. enervis*, and the distance between any plant individuals of the same plant species was more 20 m. We digged out a soil block (12 cm in diameter and 12 cm in depth) around the target plant and collected all healthy leaves (ca. ~1 g), roots (ca. ~1 g) and rhizosphere soil attached to the roots (ca. ~1 g). In total, 162 samples (3 plant species × 18 individuals × 3 compartments) were collected in this study. The samples were immediately placed in sterile plastic bags and transported to laboratory in an icebox. Root samples were washed with tap water, and the fine roots (<2 mm diameter) were picked out and washed with sterilized water. Soil samples were sieved through a 1-mm sieve to remove debris and roots. The leaf, fine root and rhizosphere soil samples were stored at −80°C until processing required for DNA extraction.

### Molecular analysis.

Genomic DNA were extracted from 0.5 g frozen leaf and root samples using the modified CTAB method, and from 0.5 g frozen soil using PowerSoil DNA isolation kit (MoBio Laboratories, Inc.) according to the instructions. The concentration of DNA was measured using a NanoDrop 1000 Spectrophotometer (Thermo Scientific). For Illumia Miseq sequencing, fungal internal transcribed spacer 2 (ITS2) of the rDNA was amplified using primers fITS7 Fun (GTGARTCATCGAATCTTTG) and ITS4 Fun (AGCCTCCGCTTATTGATATGCTTAART) (85), linked with 12-base barcode for sample distinction. The amplification was carried out in a 25 μL reaction solution containing 2.5 μL 10 × buffer, 1.5 mM MgSO4, 250 μM each dNTP, 0.75 μM each primer, 0.5 U KOD-plus-Neo polymerase (Toyobo), and 10 ng of template DNA. The PCR condition was set at 94°C for 3 min, 35 cycles for denaturation at 98°C for 30 s, annealing at 56°C for 30 s, and extension at 72°C for 40 s, followed by a final extension at 72°C for 10 min. Bacterial V4 hypervariable region of the 16S rDNA was amplified using primers F515 (GTGCCAGCMGCCGCGGTAA) and R806 (GGACTACHVGGGTWTCTAAT) (86), linked with 12-base barcode for sample distinction. The bacterial PCR amplification was carried out in a 25 μL reaction solution consisting of 2.5 μL 10 × buffer (Mg^2+^ plus), 2.5 mM each dNTP, 10 μM each primer, 1 U *Taq* DNA polymerase (TaKaRa) and ca. 10 ng of template DNA. The PCR condition was set at 95°C for 3 min, 30 cycles for denaturation at 95°C for 30 s, annealing at 58°C for 30 s, and extension at 68°C for 40 s, followed by a final extension at 68°C for 10 min. We also used sterile deionized distilled water as negative controls in all steps of the PCR procedure to test the presence of contamination in reagents. No bands were observed in any of the negative controls. The PCR products were quantified using a PCR Product Gel purification kit (Bioteke), and 50 ng purified DNA of each sample was pooled and adjusted to 10 ng μL^−1^. A sequencing library was generated by addition of an Illumina sequencing adaptor (5′-GATCGGAAGAGCACACGTCTGAACTCCAGTCACATCACGATCTCGTATGCCGTCTTCTGCTTG-3′) to the product using an Illumina TruSeq DNA PCR-Free Library Preparation Kit (Illumina), following the manufacturer’s instructions. The library was applied to an Illumina MiSeq PE250 platform for sequencing using the paired end option (2 × 250 bp) at the Environmental Genome Platform of Chengdu Institute of Biology, Chinese Academy of Sciences, China.

### Bioinformatics analysis.

For both ITS2 and 16S sequences, Quantitative Insights into Microbial Ecology (QIIME2) (87) was used for filtering raw sequences to eliminated low-quality sequences, defined as those with an average quality score < 20, without valid primer sequence or barcode sequence, containing ambiguous bases, or length < 250 bp. In addition, the fungal ITS2 region was extracted by using the fungal ITSx software package (88). The potential chimeras of ITS2 and 16S sequences were subsequently checked using the *chimera.uchime* command in MOTHUR version 1.31.2 (89) by comparison to entries in the unified system for the DNA based fungal species linked to the classification (UNITE) database (90) and bacterial species linked to the SILVA (v.12.8) database (86), respectively. The non-chimeric ITS2 and 16S sequences were clustered into operational taxonomic units (OTUs) at a 97% similarity level based on the UPRASE pipeline using USEARCH version 8.0 (91) after dereplication and discarding all singletons. A representative fungal and bacterial sequence (the most abundant) of each OTU was selected for searching against the UNITE database and SILVA database respectively using the *sintax* function (92) in USEARCH with a confidence cut-off (P) value of 0.65. In addition, the nonbacterial OTUs (chloroplast, mitochondrial, viridiplantae, and archaea sequences) were removed (93). To eliminate the effects of different sequence numbers among the samples on the analysis for fungal and bacterial communities, the number of sequences per sample was normalized to the smallest sample size of the fungal community and bacterial community using the *sub.sample* command in MOTHUR. Detailed information about fungi and bacteria in the present study is given in Table S1 and S2.

### Data analysis.

All the statistical analyses were performed in R version 3.4.4 (94). The rarefaction curves of the observed OTUs of bacteria and fungi in each plant species were calculated using the *specaccum* function in the *vegan* package (95). One-way analysis of variance (ANOVA) was used to explore the effect of compartment niche (leaf, root, and rhizosphere soil) on the OUT richness of fungi and bacteria after the log transformation as the data did not satisfy the normality of distribution or homogeneity of variance, then significant differences among compartment niches were compared using Tukey’s honestly significant difference (HSD) test at *P < *0.05. The relative abundance of abundant fungal and bacterial OTUs (top 50) among compartment niches of different plant species were depicted using the *pheatmap* function in the *pheatmap* package version 1.0.8 (96).

The distance matrices of community composition (Hellinger-transformation of the OTU read data) of fungi and bacteria were constructed by calculating dissimilarities using the Bray-Curtis method (97). Subsequently, non-metric multidimensional scaling (NMDS) was used to visualize the community dissimilarities of fungi and bacteria using the *metaMDS* command in the *vegan* package. The permutational analysis of variance (PerMANOVA) was applied to explore the effect of compartment niche on the community composition of fungi and bacteria using the *adonis* command in the *vegan* package, based on 999 permutations (95).

To show interactions of the fungal and bacterial OTUs and the distribution patterns of sensitive OTUs specific to different compartment niches, we constructed co-occurrence networks comprised both fungal and bacterial communities in each plant species according to Hartman et al. (19). We normalized the communities using the “trimmed mean of M-value” (TMM) method and expressed the values as relative abundance of count per million (CPM) in the *BioConductor* package (98), and Spearman rank correlations between OTUs were calculated. The significant correlated OTUs (Spearman's ρ > 0.7 and *P < *0.001) were visualized in networks with the Fruchterman-Reingold layout with 10^4^ permutations in the *igraph* package (99). The topological network properties such as node numbers, edge numbers, positive and negative links, average degree of co-occurrence (18), connectivity (100), and modularity (101) were calculated in the *igraph* package (99). We then analyzed the relative abundance and taxonomic position of sensitive OTUs performed within main modules of each network.

In order to screen fungal and bacterial OTUs sensitive to specific compartment niche, we tested for differential OTUs of fungi and bacteria among compartment niches based on the relative abundance of OTUs, using indicator species analysis in the *indicspecies* package (102) and likelihood ratio test in the *edgeR* package (103). Fungal and bacterial OTUs whose relative abundances were identified as significant differences among compartment niches were considered to be sensitive OTUs, using a false discovery rate (FDR) correction of *P* values (< 0.05). Furthermore, we compared the relative abundance of those sensitive OTUs in leaves to roots and in leaves or roots to soil of each plant species, and defined the OTUs with Log fold change (FC) > 1 and *P*_FDR_ < 0.05 as enriched OTUs, with Log FC < -1 and *P*_FDR_ < 0.05 as depleted OTUs, and with -1 < Log FC < 1 and *P*_FDR_ > 0.05 as no changed OTUs. Consequently, the enriched and depleted fungal and bacterial OTUs along the soil-plant continuum was visualized using a volcano map (67), and the host selection intensity was presented by depleted proportion (DP) of the fungi and bacteria calculated by the formula: the abundance of depleted OTUs (Log CPM)/the abundance of total OTUs (Log CPM) ×100. In addition, the Source Model of Plant Microbiome (SMPM) and the SourceTracker (v.1.0) based on Bayesian approach was used to estimate the sources of the bacterial and fungal communities in compartment niche of each plant species (104). The SMPM requires establishing an *a priori* model based on the known sources and relationships among microbiota of different compartment niches, and then the model was examined using SourceTracker with default parameters (26, 38).

### Data availability.

The sequencing data was uploaded to the National Center for Biotechnology Information (NCBI) (https://www.ncbi.nlm.nih.gov/) with numbers PRJNA842496 (Bacteria) and PRJNA842785 (Fungi), while the other relevant data can be found within the manuscript and its supporting materials.
