# Supplementary material for: Community Assembly of Fungi and Bacteria along Soil-Plant Continuum Differs in a Zoige Wetland
Source: Microbiol Spectr. 2022 Sep 22;10(5):e02260-22. doi: 10.1128/spectrum.02260-22 (PMC9604091; doi:10.1128/spectrum.02260-22)
Supplement: Supplemental file 4 — Fig. S1-S5, Table S4. Download spectrum.02260-22-s0005.pdf, PDF file, 1.0 MB [file spectrum.02260-22-s0005.pdf]

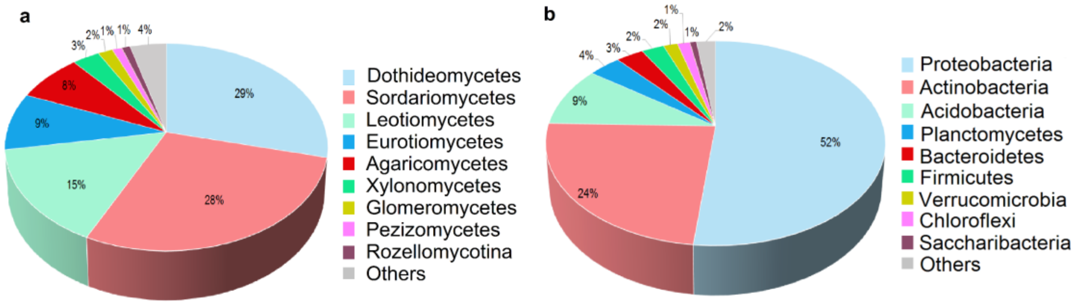

Fig. S1 Relative abundance of fungi at the class level and bacteria at the phylum level. a Fungi. b Bacteria. The fungal classes represent < 10000 of the relative abundance of the total reads, and the bacterial phyla represent < 2000 of the relative abundance of total reads are not identified to the corresponding phylum and are not identified to the corresponding classes were all assigned to “Others”.

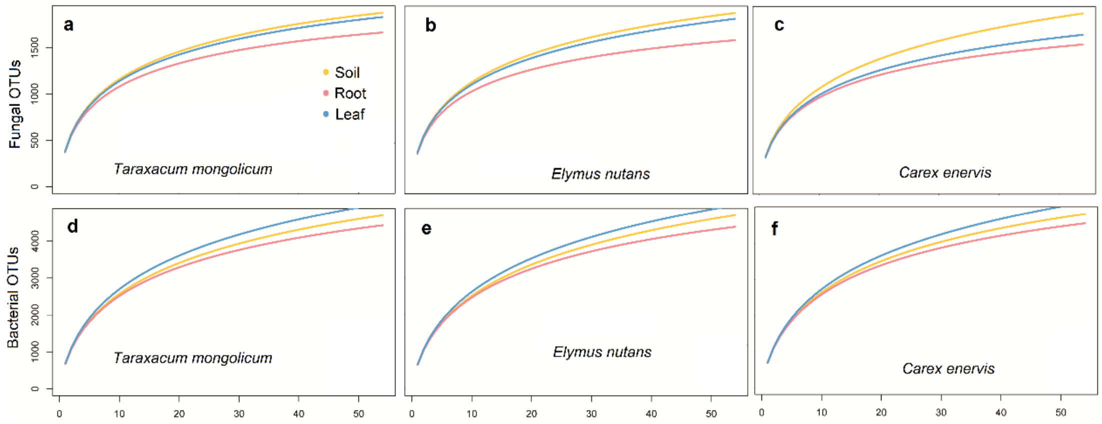

Fig. S2 The rarefaction curves of the operational taxonomic units (OTUs) of fungi and bacteria observed in soil, root and leaf of each plant species. a - c Fungi in soil, root and leaf of *Taraxacum mongolicum* (a), *Elymus nutans* (b) and *Carex enervis* (c). d – f Bacteria in soil, root and leaf of *T. mongolicum* (d), *E. nutans* (e) and *C. enervis* (f).

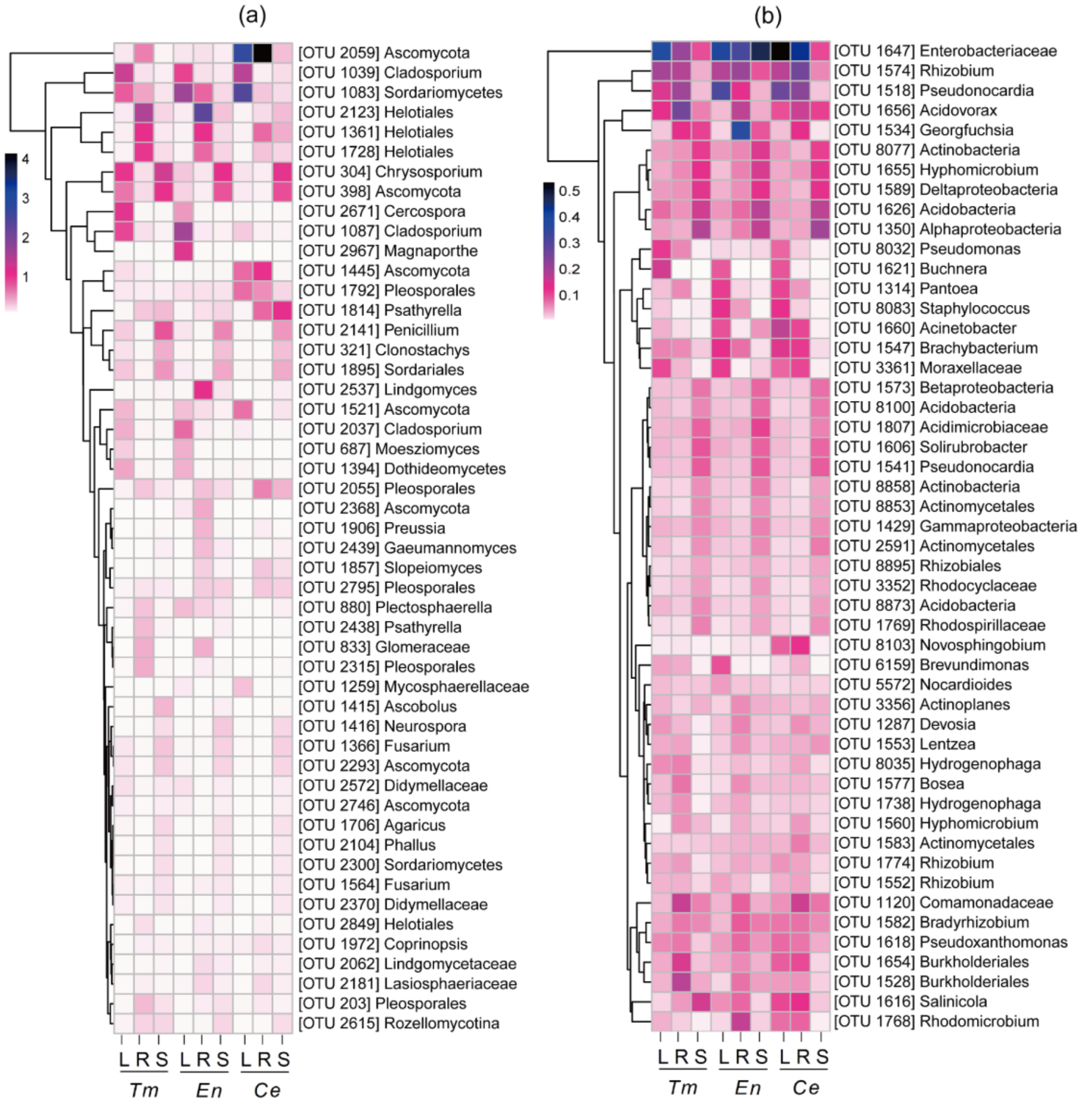

Fig. S3 Heatmap depicting the distribution of relative abundant fungal and bacterial operational taxonomic units (OTUs, top 50) associated with three plant species. a Fungi. b Bacteria. The color in each cell indicates the relative abundance of the corresponding fungal and bacterial OTUs. Cluster analysis was performed based on Bray–Curtis similarities. *Tm*: *Taraxacum mongolicum*; *En*: *Elymus nutans*; *Ce*: *Carex enervis*; L: leaf; R: root; S: soil.

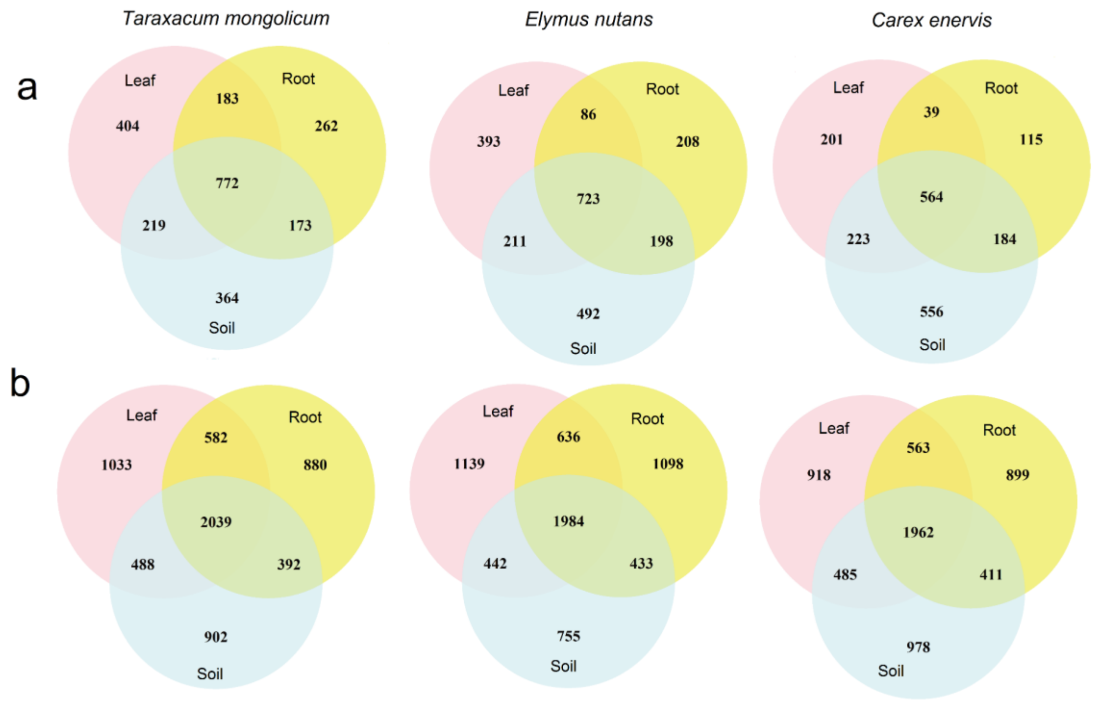

Fig. S4 The shared and exclusive operational taxonomic units (OTUs) of fungi and bacteria observed in each plant species. a Fungi. b Bacteria.

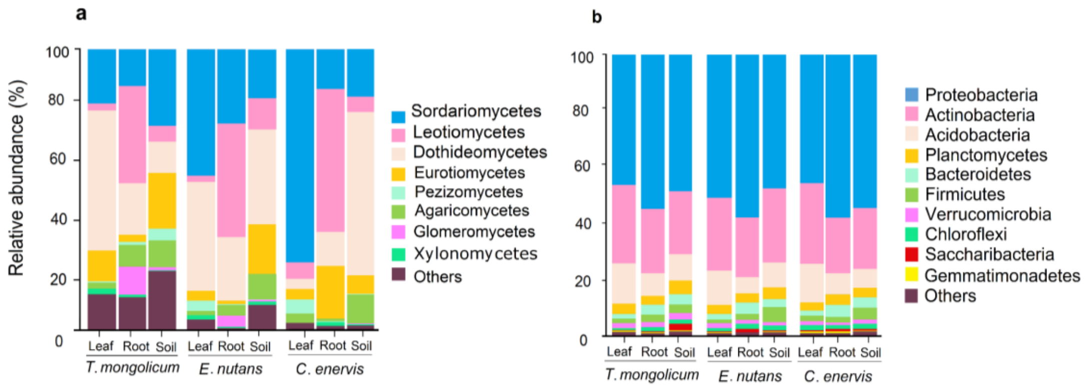

Fig. S5 Relative abundance of fungal classes and bacterial phyla associated with three plant species. a Fungi. b Bacteria. The fungal classes represent < 0.5% of the total fungal sequences and are not identified to class level, and bacterial phyla represent < 0.5% of the total bacterial sequences and are not identified to phylum level, were assigned to “Others”, respectively. *T. mongolicum*: *Taraxacum mongolicum*; *E. nutans*: *Elymus nutans*; *C. enervis*: *Carex enervis*.

Table S4 The main properties of co-occurrence networks for each plant species

Priority	*Taraxacum mongolicum*	*Elymus nutans*	*Carex enervis*
Number of nodes	935	973	1248
Number of edges	2879	2528	4384
Positive edge	2824	2301	4262
Negative edge	55	227	122
Modularity	0.649	0.735	0.425
Connectance	0.007	0.005	0.011
Average degree	6.158	5.196	9.874
Average betweenness	477.652	335.991	134.513
Average path length	10.292	5.522	3.447
Centralization degree	0.072	0.053	0.116
Assortativity degree	0.603	0.58	0.417
